# Comparison of Complications and Outcomes Following Transurethral Resection of the Prostate in Patients Presenting With and Without Acute Urinary Retention

**DOI:** 10.7759/cureus.77660

**Published:** 2025-01-19

**Authors:** Abdul Haseeb, Muhammad Zeb, Immad Ahmed, Jamal Ahmad Shah, Muhammad Moosa, Rafaqat Hussain, Muhammad Raheel, Muhammad Tayyib, Raza Muhammad

**Affiliations:** 1 Urology, Institute of Kidney Diseases, Hayatabad Medical Complex Peshawar, Peshawar, PAK; 2 General Surgery, Hayatabad Medical Complex Peshawar, Peshawar, PAK; 3 Urology, Leicester General Hospital, University Hospitals of Leicester NHS Trust, Leicester, GBR

**Keywords:** acute urinary retention (aur), benign prostatic hyperplasia, lower urinary tract symptoms, outcomes, transurethral resection of the prostate

## Abstract

Objective: The objective of this study is to evaluate the outcomes and complications of transurethral resection of the prostate (TURP) in patients with and without acute urinary retention (AUR).

Methodology: This descriptive study was conducted in the Urology Department of the Institute of Kidney Diseases (IKD), Hayatabad Medical Complex (HMC), Peshawar, from 11th August 2023 to 11th February 2024. A total of 127 male patients aged over 40 years with prostate sizes between 40 and 80 grams on ultrasonography were included. Patients with a history of prostate cancer or prior prostate surgery were excluded. All participants underwent TURP, and postoperative complications, such as urinary tract infections (UTIs), hematuria, lower urinary tract symptoms (LUTS), recatheterization, and hospital stay length, were documented. Statistical analysis was performed using IBM SPSS Statistics for Windows, Version 23 (Released 2015; IBM Corp., Armonk, New York, United States) to compare outcomes between the AUR and non-AUR groups.

Results: The study included 127 patients with a mean age of 64.92 ± 3.8 years. The incidence of AUR was 63(49.6%). Postoperative complications such as UTIs (p=0.39), hematuria (p value= 0.06), LUTS (p=0.27), recatheterization (0.52), and sepsis (0.20) were more common in the AUR group, though these differences were not statistically significant. The need for blood transfusions was also higher in the AUR group (P=0.09). Hospital stay duration and symptom resolution were comparable between the AUR and non-AUR groups.

Conclusion: AUR in benign prostatic hyperplasia patients was associated with more severe symptoms and an increased frequency of certain postoperative complications, including UTIs, hematuria, and the need for blood transfusions. However, most differences between the AUR and non-AUR groups were not statistically significant.

## Introduction

The American Urological Association (AUA) defines benign prostatic hyperplasia (BPH) as a histological condition marked by the proliferation of smooth muscle and epithelial cells in the prostatic transition zone [1]. It is a major cause of lower urinary tract symptoms (LUTS) like urgency, nocturia, frequency, dysuria, difficulty starting or emptying the bladder, and weak urine stream, and it typically affects men over 50 [1-3]. It affects nearly 210 million people globally [4,5]. Because LUTS drastically lower a person's quality of life, 30% of those who are impacted by them need surgery [3].

Symptoms of BPH include decreased urine flow, symptoms related to voiding and storage, and, most importantly, acute or chronic urine retention [4]. Medications such as alpha-blockers may be considered in select cases to alleviate symptoms or serve as an adjunct to surgical management in patients with BPH presenting with acute urinary retention (AUR) [6]. The gold standard for such patients is surgery, such as transurethral resection of the prostate (TURP), which greatly lessens the need for catheterization [4,6,7].

When undergoing TURP, patients with a history of AUR are at greater risk than those without AUR. These risks include complications, longer hospital stays, and more comorbidities [5-8]. According to a Taiwanese retrospective study, the AUR (+) group had a recatheterization rate of 13.8% (3305 men), while the AUR (-) group had none (1062 men). Additionally, the AUR (+) group had greater rates of medical costs, LUTS (22.8% vs. 16.9%), and urinary tract infections (UTIs) (18.9% vs. 15.6%) [8].

Our goal was to compare the outcomes and complications following TURP in patients with AUR and without AUR.

## Materials and methods

A descriptive study was conducted in the Urology Department of the Institute of Kidney Diseases (IKD) at Hayatabad Medical Complex, Peshawar, Pakistan, over a six-month period from August 11, 2023, to February 11, 2024. Ethical approval was obtained from the ethical department of IKD, with Reference No: 177 on July 12, 2023. A total of 127 male patients aged over 40 years were included, with prostate sizes ranging from 40 to 80 grams as determined by ultrasonography (USG).

The sample size was calculated using the WHO sample size calculator with the following formula:

n = [Z² × P × (1 - P)] / d²

Where

Z = 1.96 (for a 95% confidence interval),

P = 0.138 (expected proportion of recatheterization based on prior literature),

d = 0.06 (margin of error).

Using this formula, the calculated sample size was 127 patients. The expected proportion of 13.8% was derived from a previous study evaluating recatheterization rates in patients undergoing TURP with AUR [8].

All patients underwent TURP using a bipolar resectoscope under spinal anesthesia. Preoperative evaluations included clinical assessments, such as digital rectal examination, the International Prostate Symptom Score (IPSS), and ultrasonographic measurements of the prostate size. Patients presenting with AUR were categorized as Group A, while those without AUR were assigned to Group B. Postoperatively, patients were discharged based on stable vital signs, ability to void adequately, and no evidence of complications such as significant hematuria or infection.

Postoperative outcomes were assessed during follow-up visits conducted two weeks after surgery. Data collection focused on several parameters, including the incidence of complications such as UTIs, hematuria, sepsis, LUTS, the need for recatheterization, blood transfusion requirements, hospital stay duration, and symptom resolution, measured through changes in the IPSS.

Inclusion criteria

Patients aged above 40 to 80 years who underwent TURP surgery for BPH with a prostate size of more than 40 grams to 80 grams determined by USG irrespective of ethnicity were included.

Exclusion criteria

Patients with significant comorbidities, a history of prostate cancer, prior prostate surgeries, or other conditions causing AUR unrelated to BPH were excluded from the study.

Statistical analysis

Data analysis was performed using IBM SPSS Statistics for Windows, Version 23 (Released 2015; IBM Corp., Armonk, New York, United States). Continuous variables were expressed as means and standard deviations, while categorical variables were reported as frequencies and percentages. Comparative analyses between Groups A and B were conducted using chi-square tests for categorical variables and independent t-tests for continuous variables. Statistical significance was defined as a p-value ≤ 0.05.

## Results

A total of 127 patients were evaluated. The mean age of patients was 64.92±3.8 yrs. The mean length of hospital stay (LOS) was 3.6±0.9 days. The mean IPSS score was 13.76±7.7.

Patients were divided into two groups based on the presence of AUR. Group 1 comprised 63 patients (49.6%) who presented with AUR, while Group 2 included 64 patients (50.4%) without AUR. 

The average prostate size was 59.2 ± 8.6 grams (range 50.6 to 67.8 grams) in the AUR group and 58.7 ± 9.1 grams (49.6 to 67.8 grams) in the non-AUR group, with no statistically significant difference observed (p = 0.45) (Table 1).

**Table 1:** Baseline Clinicodemographic Variables between Two Groups An independent t-test was used for comparing the mean of these variables.

Baseline Clinicodemographic Variables		AUR (n=63)	Non-AUR (n=64)	p-Value
Age (years)		65.1 ± 4.0	64.8 ± 3.7	0.39
Prostate size (grams)		59.2 ± 8.6	58.7 ± 9.1	0.45

**Figure 1:**
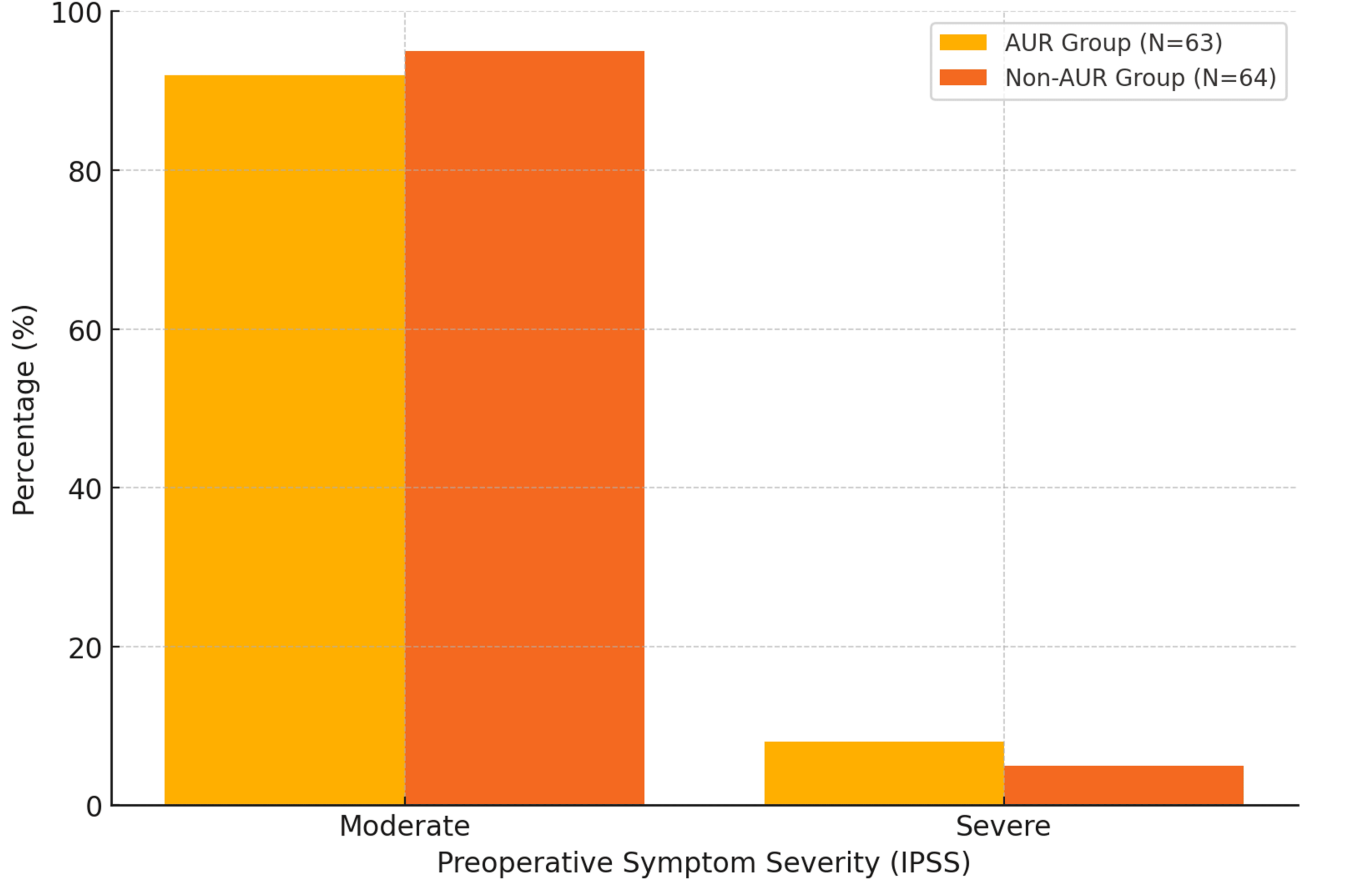
Preoperative Symptom Severity between Two Groups AUR: Acute Urinary Retention; IPSS: International Prostate Severity Score

Preoperative symptom severity was classified as "moderate" for the majority of patients in both groups, with 58 (92%) AUR patients and 61 (95%) non-AUR patients falling into this category. Severe symptoms were reported in five (8%) AUR patients and three (5%) non-AUR patients. The difference in preoperative symptom severity between the groups was not statistically significant (p = 0.32), indicating that both groups were comparable in terms of baseline clinical characteristics (Figure 1).

**Figure 2:**
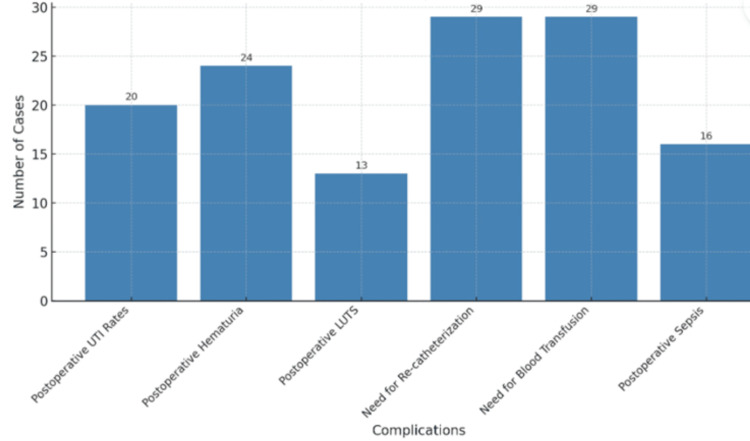
Overall Postoperative Complications LUTS: Lower Urinary Tract Symptoms

Overall postoperative complications are summarized in the bar chart (Figure 2).

According to Table 2, postoperative UTIs occurred at similar rates in both groups, affecting 11 (17.5%) patients in the AUR group and nine patients (14.1%) in the non-AUR group (p = 0.39). Hematuria was more frequently observed in 16 patients (25.0%) in the non-AUR group compared to eight (12.7%) in the AUR group, though the difference did not reach statistical significance (p = 0.06). LUTS were reported by eight (12.7%) AUR patients and five (7.8%) non-AUR patients (p = 0.27). The need for recatheterization was comparable between the groups, with 14 (22.2%) AUR patients and 15 (23.4%) non-AUR patients requiring the procedure (p = 0.52). Blood transfusions were more frequently required in the AUR group i.e. 18 (28.6%) than in the non-AUR group 11 (17.2%), although the difference was not statistically significant (p = 0.09). Sepsis occurred in 10 (15.9%) AUR patients compared to six (9.4%) non-AUR patients, with no significant difference observed (p = 0.20). Symptom resolution was achieved in 55 (87.3%) of patients in the AUR group and 61 (95.3%) in the non-AUR group, a difference that was not statistically significant (p = 0.10). However, a significantly higher proportion of severe IPSS cases were observed in the AUR group i.e. 22 (34.9%) compared to the non-AUR group i.e. 2 (3.1%). Conversely, all mild cases were found in the non-AUR group i.e. 33 (51.6%), resulting in a significant difference (p < 0.001). Regarding hospital stay duration, a greater proportion of patients in the non-AUR group had shorter stays (<3 days) at 39 (60.9%), compared to 33 (52.4%) in the AUR group, though the difference was not statistically significant (p = 0.21).

**Table 2:** Comparison of Postoperative Outcomes between AUR and Non-AUR Groups UTIs: Urinary Tract Infections; LUTS: Lower Urinary Tract Symptoms; IPSS: International Prostate Severity Score Comparative analyses between Groups A and B were conducted using chi-square tests for categorical variables.

Outcomes	Category	AUR Group	Non-AUR Group	Total	p-Value
Postoperative UTI	Yes	11 (17.5%)	9 (14.1%)	20	0.39
	No	52 (82.5%)	55 (85.9%)	107	
Postoperative Hematuria	Yes	8 (12.7%)	16 (25.0%)	24	0.06
	No	55 (87.3%)	48 (75.0%)	103	
Postoperative LUTS	Yes	8 (12.7%)	5 (7.8%)	13	0.27
	No	55 (87.3%)	59 (92.2%)	114	
Need for Re-catheterization	Yes	14 (22.2%)	15 (23.4%)	29	0.52
	No	49 (77.8%)	49 (76.6%)	98	
Need for Blood Transfusion	Yes	18 (28.6%)	11 (17.2%)	29	0.09
	No	45 (71.4%)	53 (82.8%)	98	
Postoperative Sepsis	Yes	10 (15.9%)	6 (9.4%)	16	0.20
	No	53 (84.1%)	58 (90.6%)	111	
Resolution of Symptoms	Yes	55 (87.3%)	61 (95.3%)	116	0.10
	No	8 (12.7%)	3 (4.7%)	11	
IPSS	Mild	0 (0.0%)	33 (51.6%)	33	0.001
	Moderate	41 (65.1%)	29 (45.3%)	70	
	Severe	22 (34.9%)	2 (3.1%)	24	
Length of Hospital Stay	<3 Days	33 (52.4%)	39 (60.9%)	72	0.21
	>3 Days	30 (47.6%)	25 (39.1%)	55	

## Discussion

In our study, 127 patients with BPH were analyzed, with a mean age of 64.92 ± 3.8 years and an LOS of 3.6 ± 0.9 days. The mean IPSS was 13.76 ± 7.7, reflecting a moderate symptom burden among the cohort. Although the difference in the prostate size between the AUR and non-AUR groups was minimal (0.5 grams) and not statistically significant, its clinical relevance warrants consideration. However, given the negligible size difference observed in our study, it is unlikely to have had a meaningful impact on outcomes in this cohort.

AUR was observed in 49.6% of the patients, while 50.4% did not experience retention. This prevalence aligns with findings by Addepalli et al., who reported similar AUR rates in BPH patients [9]. AUR was more prevalent among individuals aged 60-80 years (58 cases) compared to those aged 40-59 years (five cases), consistent with that reported by Cathcart et al., who identified advanced age as a significant risk factor for AUR [10].

Postoperative urinary tract infections (UTIs) occurred slightly more frequently in the retention group (17.4%; 11 cases) than in the non-retention group (14.06%; nine cases), but the difference was not statistically significant (p = 0.39). These results are comparable to those of Chen et al., who reported UTI rates of 18.6% in the AUR group and 15.6% in the non-AUR group [8]. Conversely, Mebust et al. observed significantly lower UTI rates at 3.9% [11].

Postoperative hematuria was more frequent in the non-retention group (25.0%; 16 cases) compared to the retention group (12.7%; eight cases), though this trend was not statistically significant (p = 0.06). This contrasts with Ramachandraiah et al., who reported higher hematuria rates in AUR patients [3]. Similarly, postoperative LUTS were reported in eight cases in the retention group and five cases in the non-retention group (p = 0.27), consistent with Ramachandraiah et al., who found no significant differences in LUTS between these groups [3].

The need for recatheterization was nearly identical between groups, with 14 cases in the retention group and 15 in the non-retention group (P = 0.52). These findings differ from those of Ramachandraiah et al., Chen et al., Mebust et al., and Mahmood et al., who reported a significant impact of AUR on recatheterization rates [3,8,11,12]. Sepsis rates were slightly higher in the AUR group (15.9%; 10 cases) than in the non-AUR group (9.4%; six cases), but the difference was not statistically significant (p = 0.20). This aligns partially with that by Ramachandraiah et al., who reported 2.1% sepsis in AUR patients and none in the non-AUR group [3], while Chen et al. observed sepsis in 1.4% of AUR patients [8]. In contrast, Mebust et al. and Mahmood et al. reported minimal to no sepsis cases, highlighting the variability in outcomes across studies [11, 12]. Although specific contributing factors were not analyzed in this study, existing literature suggests that incomplete bladder emptying, detrusor underactivity, and postoperative complications, such as urethral edema or infection, may play a role [3,8,11,12]. Additionally, surgical factors, such as suboptimal resection or intraoperative complications, and severe preoperative symptoms (e.g., high IPSS scores) have been associated with recatheterization in other studies. Management of such cases generally involves short-term recatheterization to allow recovery of bladder function, the use of alpha-blockers to improve urinary flow, and follow-up evaluations to reassess voiding ability. Persistent cases may require additional interventions, such as repeat procedures or further imaging to identify underlying issues.

Symptom resolution was slightly lower in the retention group (87.3%; 55 cases) compared to the non-retention group (95.3%; 61 cases), but the difference was not statistically significant (p = 0.10). These findings are consistent with those by Chen et al., who observed similar rates of symptom resolution between the groups [8]. Immediate transurethral surgery after AUR episodes has been shown to improve voiding and catheter-free outcomes with extensive prostate tissue resection, though a significant proportion of patients (46.8%) required alpha-blockers three months postoperatively [13]. The requirement for alpha-blockers in postsurgery suggests the presence of residual LUTS despite surgical intervention. This could be attributed to a variety of factors, including incomplete resolution of bladder outlet obstruction, detrusor muscle dysfunction, or persistent inflammation. In some cases, pre-existing bladder overactivity may continue to contribute to symptomatology, necessitating pharmacological support. Furthermore, alpha-blockers are often used in the postoperative period to improve urinary flow and alleviate irritative symptoms, particularly during the recovery phase. These findings highlight the need for individualized postoperative management strategies and further research to evaluate the predictors of prolonged medication use after TURP.

The AUR group demonstrated a higher, though not statistically significant, need for blood transfusions (28.6% vs. 17.2%, P = 0.09). Studies such as those by Chen et al. have suggested a potential link between AUR and increased intraoperative blood loss [8]. A meta-analysis also identified a significantly higher risk of intraoperative blood transfusions in the urinary retention group compared to those with lower urinary tract symptoms alone (RR: 1.90, P = 0.002), potentially due to increased bladder wall tension or more extensive surgical dissection [14].

Hospital stay durations were similar between groups, with a slightly higher proportion of non-AUR patients discharged within three days (60.9% vs. 52.4%, P = 0.21). These findings align with studies by Chelliah et al. and Pogula et al., which suggest that hospital stay is more closely associated with surgical complexity than AUR status [15,16]. Innovative surgical techniques, such as HoLEP and GreenLight PVP, have been associated with shorter hospital stays compared to Monopolar-TURP, though the clinical significance of these differences remains limited [6].

Limitations

This study has several limitations. It was conducted at a single center with a small sample size, limiting generalizability and statistical power. The short follow-up period of two weeks may not capture long-term outcomes or complications. Excluding patients with significant comorbidities or non-BPH causes of AUR narrows the applicability of the findings. The descriptive, non-randomized design makes it susceptible to selection bias, and potential confounders such as the extent of prostate resection and surgeon experience were not controlled. Additionally, only bipolar TURP was analyzed, without comparison to other techniques. Trends in complications were often not statistically significant, and data collection relied on follow-up visits, potentially underreporting complications. These limitations underscore the need for larger, multicenter studies with longer follow-ups to confirm and expand these findings.

## Conclusions

In conclusion, AUR in BPH patients was associated with more severe symptoms and certain postoperative trends such as higher rates of postoperative UTIs, hematuria, and blood transfusions. While the AUR group did exhibit trends toward more severe complications, none of the differences were statistically significant.
